# The Genus *Eranthis*: Prospects of Research on Its Phytochemistry, Pharmacology, and Biotechnology

**DOI:** 10.3390/plants12223795

**Published:** 2023-11-07

**Authors:** Andrey S. Erst, Natalia V. Petrova, Olga A. Kaidash, Wei Wang, Vera A. Kostikova

**Affiliations:** 1 Central Siberian Botanical Garden, Siberian Branch of Russian Academy of Sciences (CSBG SB RAS), Novosibirsk 630090, Russia; erst_andrew@yahoo.com; 2 Komarov Botanical Institute, Russian Academy of Sciences (BIN RAS), St. Petersburg 197022, Russia; npetrova@binran.ru; 3 Central Research Laboratory, Siberian State Medical University, Tomsk 634050, Russia; kaidash_2011@mail.ru; 4 State Key Laboratory of Systematic and Evolutionary Botany, Institute of Botany, Chinese Academy of Sciences, Beijing 100093, China; wangwei1127@ibcas.ac.cn; 5 College of Life Sciences, University of Chinese Academy of Sciences, Beijing 100049, China

**Keywords:** *Eranthis*, chromone, furochromone, triterpene saponin, coumarin, biological activity, biotechnology

## Abstract

This review summarizes information about the chemical composition and beneficial properties of species of the genus *Eranthis* Salisb. from the world’s flora. To date, seven out of ~14 species found in Asia and parts of Europe have been studied to various degrees. Here, data are presented on the diversity of sets of chromones, furochromones, triterpene saponins, coumarins, and other classes of secondary metabolites of *Eranthis* species according to the literature. For new compounds—isolated from *Eranthis* for the first time—structural formulas are also provided. Among the new compounds, chromones and coumarins predominate, as do triterpene saponins of the olean and cycloartane series and lectin. The results of pharmacological studies are presented showing anti-inflammatory, antioxidant, antiviral, and other types of biological activities found in extracts, in their fractions, and in individual compounds of the aboveground and underground organs and parts of *Eranthis* species. Despite the limited geographic range of *Eranthis* plants, it is possible to search for active substances, develop methods for biological and chemical synthesis of the isolated substances, and create a finished therapeutic substance based on them. In addition, it is feasible to obtain the desired standardized pure materials from *Eranthis* species grown in vitro.

## 1. Introduction

**Figure 1:**
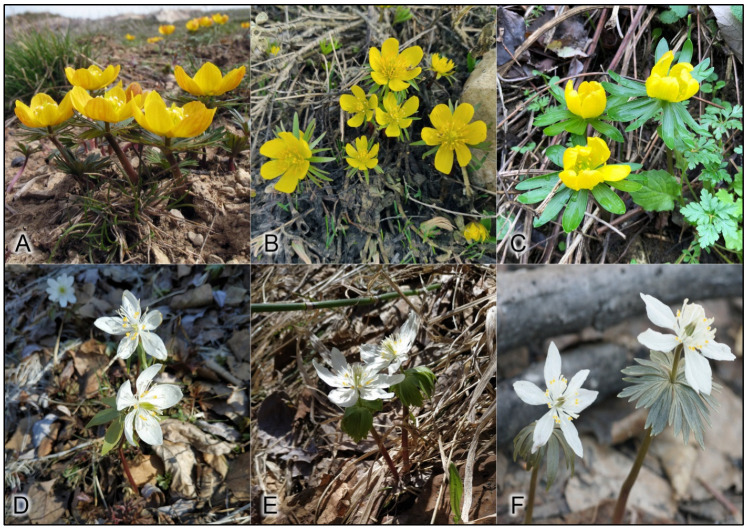
Species of the genus *Eranthis*. (**A**) *E. longistipitata*, (**B**) *E. cilicica*, (**C**) *E. hyemalis*, (**D**) *E. sibirica*, (**E**) *E. tanhoensis*, and (**F**) *E. stellata*.

According to molecular and morphological data, the tribe Cimicifugeae Torrey & Gray belongs to the family Ranunculaceae Juss. and includes four recognized genera and ~49 species: *Actaea* L. (32 species), *Anemonopsis* Siebold et Zucc. (one species), *Beesia* Balf. f. et W. W. Sm. (two species), and *Eranthis* Salisb. (14 species) [1,2,3]. Most of these species occur mainly in the northern hemisphere and are perennial herbs [4]. The taxonomic position of the genera *Eranthis* and *Beesia* has been a matter of systematic uncertainty within the tribal rank in the Ranunculaceae family. According to morphological information, *Beesia* has been assigned to three different tribes (Helleboreae DC., Actaeeae Spach, and Trollieae Schröd.) by intuitive taxonomic techniques but has seldom been included in cladistic analyses [5,6,7]. By contrast, the *Eranthis* genus has consistently been assigned to the Helleboreae tribe or as the only genus to the tribe Eranthideae T. Duncan & Keener in morphological classifications but always has been a sister taxon to plants of the Actaeeae tribe in cladistic analyses [8]. The genus *Eranthis* consists of 8–14 species growing in southern Europe and temperate Asia [9,10,11]. Traditionally, the genus has been subdivided into two sections: *Eranthis* sect. *Eranthis* and *E.* sect. Shibateranthis (Nakai) Tamura [12]. The type section *Eranthis* is characterized by plants with tubers, yellow sepals, and emarginate or slightly bilobate upper petal margins without pseudonectaries (Figure 1) [6,11]. The section *Eranthis* in Europe includes *E. hyemalis* (L.) Salisb. and *E. bulgarica* (Stef.) Stef., whereas in Southwest and West Asia, it includes *E. cilicica* Schott et Kotschy, *E. kurdica* Rukšāns, *E. longistipitata* Regel, and *E. iranica* Rukšāns et Zetterl. [13,14,15,16]. The section Shibateranthis has long-lived tubers, white sepals, and bilobate or forked petal margins with pseudonectaries (Figure 1) [6,17]. Representatives of this section have a natural geographic range in temperate North and East Asia (*E. albiflora* Franch., *E. byunsanensis* B.Y. Sun, *E. lobulata* W.T.Wang, *E. pinnatifida* Maxim., *E. pungdoensis* B.U. Oh, *E. sibirica* DC., *E. stellata* Maxim., and *E. tanhoensis* Erst) [10,11].

Plants of the tribe Cimicifugeae are some of the richest sources of various active ingredients and of therapeutic and health-promoting substances. The value of the constituents has been confirmed by many years of use in East Asian countries in folk medicine. Thus, it is important to integrate new technologies into research on Cimicifugeae, both for the sustainable use of pharmaceutical resources from Cimicifugeae and for a search for new compounds with potential clinical efficacy and fewer adverse effects [18,19,20]. In the tribe Cimicifuga, representatives of the genus *Actaea* are the most frequently studied plants in the world of science. Nonetheless, little is known about the chemical profile and biological activity of other representatives of Cimicifugeae: *Beesia* and *Anemonopsis*. In recent decades, new information has been obtained about the chemical profiles of (and biological effects of extracts and individual compounds from) *Eranthis* species, which are early flowering geophytes with a limited geographic range. No reviews on plants of this genus have previously been carried out to the best of our knowledge. In this brief review, we summarize research on the phytochemistry, pharmacology, and biotechnology of *Eranthis* plants of the world flora since 1961.

## 2. Phytocomponents Identified in *Eranthis* Plants and Their Chemotaxonomic Significance

### 2.1. Chromones

Since the 1960s, from some *Eranthis* species, a series of substances has been isolated that represents an important class of oxygen-containing heterocyclic compounds that are derivatives of benzo-γ-pyrone: chromones. Their isolation has been performed by various chromatographic methods, and the structures of individual compounds have been investigated by 1-dimensional (H-NMR) and 2-dimensional nuclear magnetic resonance (C-NMR) spectroscopy. In structure, chromones are similar to flavonoids and coumarins but are substantially less common in the wild. Chromones can give rise to hydroxy- and methoxy-derivatives and can attach a sugar moiety, whereas after condensation with benzene, pyran, or furan rings, they can be transformed into a variety of benzo-, pyrano-, or furochromones, respectively. Compounds from the class “simple furochromones and chromones” are most often found in *Eranthis* species; chromones have been detected in underground parts, whereas furochromones have been found in underground and aboveground parts (Table 1).

**Figure 2:**
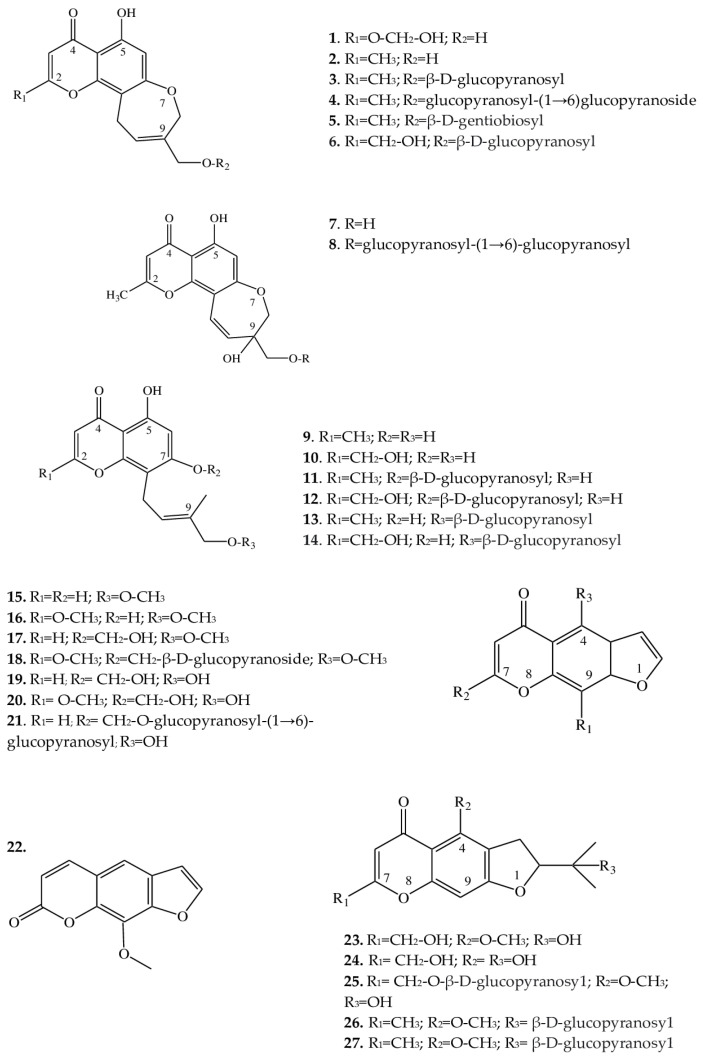
Structures of furochromones and chromones from *Eranthis* species.

Structurally, the chromones found in *Eranthis* species can be categorized into several classes (Figure 2). Structures of compounds **1**–**6** are similar in the carbon backbone—containing an oxepin ring—but differ from one another in substituents at positions C-2 and C-9. These substituents can be methyl and hydroxymethyl groups as well as mono- and diglycosides. These compounds have been registered in the underground parts of *E. cilicica* Schott & Kotschy and *E. hyemalis* (L.) Salisb. [21,22,23]. The first publications on the isolation of chromones of this subclass date back to the end of the 1970s, when 5-hydroxy-9-hydroxymethyl-2-methyl-8,11-dihydro-4H-pyrano[2,3-g][1]benzoxepin-4-one [named eranthin (**2**)] and its β-D-glucoside (**3**) were isolated [22].

Structures similar to this type (**7** and **8**), in contrast to the above compounds, have a displaced double bond [from C-9(10) to C-10(11)] on the oxepin ring; in addition, at position C-9, there is both an oxymethylene group bearing various substituents and a hydroxyl group. These compounds have so far been found only in the underground part of *E. cilicica* [21]. ^1^H and ^13^C nuclear magnetic resonance data have allowed us to identify the structural features of compounds **9**–**14** and to determine that their oxepin ring is open; a 4′-hydroxy-3′-methylbut-2′-enyl group has been found at position C-8. Compounds **11** and **12** additionally contain a D-glucose residue at the C-7 position, whereas compounds **13** and **14** contain it at the C-4′ position. These chromones have been detected in the underground parts of *E. cilicica* and *E. hyemalis* [21,22]. All of the above chromones are specific to the genus *Eranthis*.

### 2.2. Furochromones

These compounds of *Eranthis* species are formed by the condensation of a simple chromone with a furan ring at positions C-6 and C-7 and, in contrast to the aforementioned chromones, are relatively common in the plant kingdom. For instance, the first representative of this subclass of compounds called khellin (**16**) has long been used in folk medicine to relieve ureteral pain during colic. For the first time, khellin was found in a seed extract of *Ammi visnaga* (L.) Lam. and was isolated as far back as the end of the 19th century [36]. Currently, khellin’s ability to act directly on smooth-muscle fibers is widely used in clinical practice [37]. Khellin, aside from species of the genus *Ammi*, has been found in other representatives of the family Apiaceae Lindl., for example, in *Dioscorea* L. sp. and *Pimpinella* L. sp. [38,39,40], and among *Eranthis* species, in *E. hyemalis* and *E. longistipitata* Regel [24,25]. The diversity of the structures in the furochromone subclass, which includes khellin, is mostly determined by the presence of substituents at the C-4, C-7, and C-9 positions. At the C-4 position, methoxy or hydroxyl groups can serve as a substituent; at position C-7, methoxy groups and glucose; and at position C-9, a methoxy group, or—as in **15**, **17**, **19**, and **21**—the substituent may be absent. Khellol (**17**) represents an aglycone of khellol glucoside (**18**), in which the sugar moiety is attached at position C-7. In the genus *Eranthis*, most research on furochromones of this subclass has been conducted on samples of the aerial parts (leaves, stems, and flowers) of *E. pinnatifida* Maxim., *E. hyemalis*, and *E. longistipitata* [24,25,26,27], and only compound **21** has been detected in an underground part (tubers) of *E. cilicica* [21].

Recently, new compounds not previously found in *Eranthis* species were discovered in samples of *E. longistipitata* from Central Asia (Kyrgyzstan): methoxsalen (**22**), 5-*O*-methylvisammioside (**26**), and visamminol-3′-*O*-glucoside (**27**) [25]. Methoxsalen (**22**) is often seen in the plant extracts of such families as Apiaceae, Rutaceae Juss., Fabaceae Lindl., and Brassicaceae Burnett [41,42], whereas the last two compounds of this subclass (**26** and **27**) have been registered only in extracts from an underground part of *Saposhnikovia divaricata* (Turcz.) Shischk. (Apiaceae) [43,44,45].

Because *Eranthis* species synthesize chromones during normal physiological processes, preliminary conclusions have been made that the genus *Eranthis* is closest to the genera *Cimicifuga* and *Actaea* (in whose extracts, chromones have also been found), and not *Helleborus* L. (for example), whose species do not synthesize chromones but are distinguished by the accumulation of cardenolides and bufadienolides [23,46].

### 2.3. Triterpene Saponins

A phytochemical analysis of a methanol extract from tubers of *E. cilicica* has revealed two new bisdesmosidic triterpenes, named eranthisaponins A (**28**) and B (**29**) [28]. The new saponins are based on the structural backbone of hederagenin, which is a triterpenoid first isolated from seeds and leaves of *Hedera helix* L. [47]. A distinctive feature of eranthisaponin A (**28**) is a branched tetraglycoside attached at the C-3 position of the aglycone, whereas a feature of eranthisaponin B (**29**) is a linear hexaglycoside attached at the C-28 position of the aglycone. Such sugar forms in triterpene saponins have not been described previously. In addition, as one of the substituents, eranthisaponin A (**28**) contains D-allopyranose: a monosaccharide that is extremely rare in plant saponins [28].

Furthermore, in *Eranthis* plants, a number of known triterpene saponins (**30**–**35**) have been discovered that are (just as eranthisaponins B and A) based on the backbone of hederagenin with substituents at positions C-3 and C-28; extremely rarely (only in **31**), the substituent (a hydroxyl group) is located at the C-23 position. Other substituents include di- and triglycosides composed of glucose, arabinose, and rhamnose residues.

The research continued by K. Watanabe with coauthors [29] has allowed to subsequently isolate a new oleanane glycoside (**34**) from the tubers of *E. cilicica*. Another oleanane glycoside (**35**) had been discovered earlier in the underground part of *Anemone coronaria* L. (Ranunculaceae) [48]. These substances (**34** and **35**) contain triglycosides only at position C-3 of the carbohydrate part of the molecule. All of the above triterpene saponins belong to the oleanan type.

**Figure 3:**
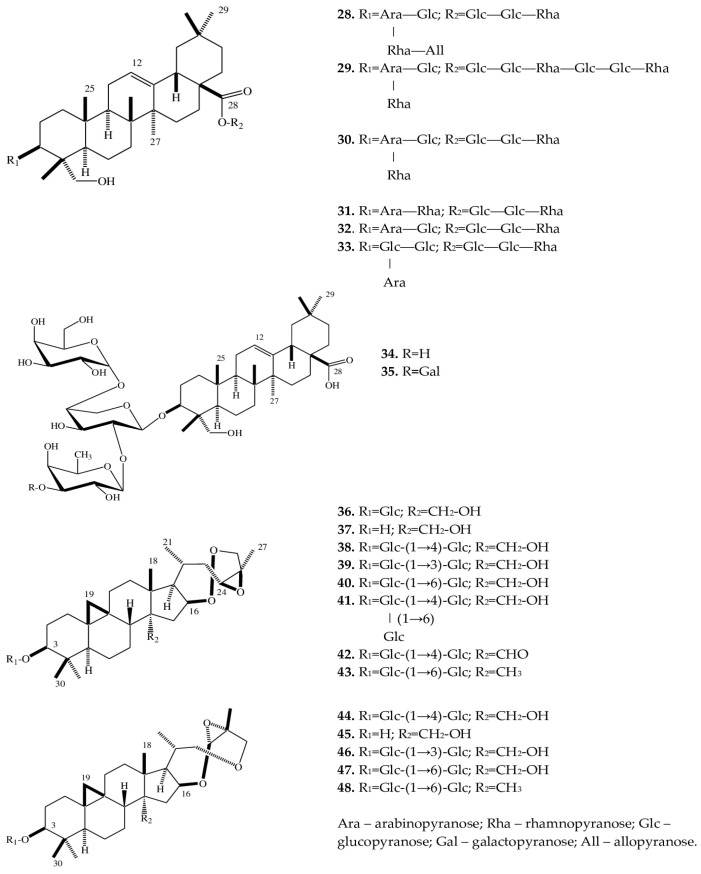
Structures of triterpene saponins of *Eranthis* species.

In the same study [29], when fractionating a methanol extract from the tubers of *E. cilicica*, Watanabe et al. isolated several cycloartane-type compounds (**36**–**48**). There were 13 such triterpene saponins, all of which had not been characterized before. Compounds **37** and **45** are aglycones of **36** and **44**, respectively. The new compounds can be categorized into two very similar subclasses: **36**–**43** and **44–48**. In terms of their structure, rings A–D are similar, and differences lie in rings E and F (Figure 3).

The compounds of both subclasses differ among themselves in the presence of various sugar moieties at the C-3 position of the aglycone as well as in the presence of a hydroxy, oxo, or methyl group at position C-28.

### 2.4. Alkaloids

In the tubers and aerial parts of *E. hyemalis*, trace amounts of an alkaloid called corytuberine (**49**) have been found [30].

### 2.5. Coumarins

In ongoing studies on *E. longistipitata*, coumarins have been discovered in aqueous-ethanol extracts from the leaves of this species: this class of compounds was registered in the genus *Eranthis* for the first time [25] but is widespread in the family Ranunculaceae [49]. 5,7-Dihydroxy-4-methylcoumarin (**50**), scoparone (**51**), and fraxetin (**52**) are affiliated with the subclass “simple coumarins”, which are based on a coumarin molecule with substituents in the form of methyl, hydroxy, and methoxy groups. Luvangetin (**53**) can be assigned to linear pyranocoumarins, in which—aside from various substituents—a pyran ring is present in the backbone.

### 2.6. Flavonoids

In contrast to the sets of chromones and triterpene saponins, the set of flavonoids in *Eranthis* species mainly contains known substances. For instance, a study on the aqueous-ethanol extracts from the leaves of four *Eranthis* species has led to the identification of several flavonoids: quercetin (**54**) (*E. longistipitata*, *E. stellata* Maxim., and *E. tanhoensis*), kaempferol (**62**) (*E. longistipitata*, *E. stellata*, and *E. tanhoensis*), vitexin (**66**) (*E. sibirica* DC.), and orientin (**67**) (*E. sibirica* and *E. stellata*) [31]. In addition, in *E. longistipitata*, from the class of flavonols, researchers have identified isoquercitrin (**55**), hyperoside (**56**), reynoutrin (**57**), quercetin-3-sambubioside (**59**), peltatoside (**60**), rutin (**61**), juglalin (**63**), and trifolin (**64**); from flavanones, aromadendrin (**65**) and 6-methoxytaxifolin (**71**); from C-glycoside flavones, there is carlinoside (**68**); from the class of flavans, investigators have identified cianidanol (**69**) and auriculoside (**70**); and from chalcones, aspalathin (**72**), phloridzin (**73**), and phloretin (**74**) [32]. Flavonoids in the leaves of *E. hyemalis* are represented by the glycosides of quercetin and kaempferol, the detailed structures of which have not been determined [27]. The heterogeneity of the qualitative and quantitative profiles of flavonoids has been noted among the analyzed *Eranthis* species [11,31,32].

### 2.7. Phenolcarboxylic Acids

The investigation of this class of phenolic compounds is represented by a single publication covering only three *Eranthis* species and dealing with the identification of phenolcarboxylic acids that are widespread in nature: chlorogenic (**75**) (*E. sibirica*, *E. stellata*, and *E. tanhoensis*), caffeic (**76**) (*E. sibirica* and *E. stellata*), salicylic (**77**) (*E. sibirica* and *E. tanhoensis*), and gentisic (**78**) (*E. stellata*), whereas the concentration of caffeic acid (0.29–0.32 mg/g), chlorogenic acid (0.34–0.96 mg/g), and salicylic acid (0.25 mg/g) has proven to be the highest in *E. sibirica* [31]. Thus, there is evidence of variation in the profile and levels of phenolcarboxylic acids among these species [11,31,32].

### 2.8. Fatty Acids

To date, fatty acids in the leaves of *E. longistipitata* (**82**, **84**, **88**–**98**, and **104**) and the composition of seed oil from *E. hyemalis* (**79**–**81**, **83**, **85**–**87**, **91**, **99**–**103**, and **105**–**111**) have been determined [25,33].

Although the chemical composition of seed oil has been investigated only in *E. hyemalis*, this class of compounds deserves special attention because in most other genera of Ranunculaceae it is taxonomically significant. For instance, in most of *Ranunculus* L. species, hexadecadienoic acid (16:2n − 6) is dominant and constitutes 2–10% of seed oil. For the genera *Pulsatilla* Mill., *Adonis* L., and *Aconitum* L. and some *Anemone* L. species, the major fatty acid (up to 80% of total) is linoleic acid, whereas the relative abundance of eicosadienoic acid reaches 7–8% in some *Anemone* species, and its concentration in the species of *Cimicifuga* Wernisch., *Helleborus* L., *Actaea* L., and *Caltha* L. is the lowest [50]. In *E. hyemalis*, *cis*-13,16-docosadienoic acid (**109**) serves as a major fatty acid, constituting up to 57% of seed oil [33]. In terms of the total set of fatty acids in seed oil, *E. hyemalis* is close to the genera *Cimicifuga* and *Actaea*, but more detailed conclusions require additional investigation.

### 2.9. Lectins

The name lectin was proposed by W. Boyd in 1954 [51] for proteins that can agglutinate red blood cells and selectively bind to carbohydrates [52]. So far, more than 500 lectins have been isolated from higher and lower plants [53] and can accumulate in roots, leaves, fruits, seeds, and wood [54,55]. It is believed that they provide protection to plants from phytopathogenic microorganisms and phytophages, play a decisive role in the establishment of symbiotic relationships with nitrogen-fixing bacteria, and participate in the transport of hormones and glycoproteins [55,56,57].

To date, in the family Ranunculaceae, only in *Clematis montana* Buch.-Ham and *Eranthis hyemalis* have researchers demonstrated the presence of lectins. The *E. hyemalis* lectin, called EHL, was first isolated in the second half of the 1980s by B.P. Cammue [34]. This lectin represents the most widespread type of lectin among plants: ribosome-inactivating proteins [58,59,60]. EHL, just like other ribosome-inactivating proteins, consists of two chains: chain A is responsible for enzymatic activity, and chain B binds carbohydrates, thereby helping the lectin molecule get inside the cell. In terms of its specificity to carbohydrates, EHL belongs to type II, that is, it can bind to D-galactose and N-acetyl-D-galactosamine. The role of bound carbohydrates is probably to increase the water solubility of a given glycoprotein [61]. Structurally, EHL resembles the lectin of *Bryonia dioica* Jacq. (Cucurbitaceae), but the latter possesses moderate activity, its relative abundance does not exceed 0.4% of the total soluble protein, and this lectin is found in all vegetative organs [62]. On the contrary, EHL is located in underground organs, its relative abundance reaches 2% of the total amount of soluble proteins, and in terms of activity, EHL exceeds the lectin of *B. dioica* 20-fold [34]. The research of B.P. Cammue was expanded in 1993 by M.A. Kumar and coworkers, who characterized in detail physicochemical properties of the lectin and determined a part of amino acid sequence of chain A [35].

### 2.10. Compounds from Other Classes

In a leaf extract of *E. longistipitata*, by means of liquid chromatography combined with high-resolution mass spectrometry, the presence of amino acid–related compounds (**112**–**117**) has been established: D-(+)-pyroglutamic acid, D-(+)-tryptophan, isoleucine, L-phenylalanine, L-tyrosine, and D-(−)-glutamine; the researchers detected organic acids (**118**–**120**): citric acid, D-α-hydroxyglutaric acid, and gluconic acid; sugars (**121**–**123**): α-lactose, D-(+)-galactose, and α-trehalose; alcohol D-(−)-mannitol (**124**); and phenylpropanoid 6-gingerol (**125**) [25].

## 3. Prospects for the Pharmaceutical Use of *Eranthis* Plants

*Eranthis* species are not well investigated from phytochemical and pharmaceutical points of view, most likely owing to their limited endemic occurrence. Nevertheless, there are substantial prospects for the practical application of these species because plants of the tribe Cimicifugeae are some of the richest sources of various biologically active compounds as well as health-promoting and therapeutic agents [18,20]. There is evidence that an entire *Eranthis* plant can be applied against urolithiasis and diuresis [63]. The analysis of the literature data on biological activity of *Eranthis* plants showed that there are few in vitro studies on extracts from the plants of this genus (Table 2). In vivo studies on these plants have not been conducted yet; hence, they are interesting and topical for further investigation.

In in vitro experiments, an ethanolic extract from the roots of *E. hyemalis* manifests moderate inhibitory activity toward cyclooxygenase 1 (COX-1) (half-maximal inhibitory concentration [IC_50_] = 49.40 μg/mL) and toward COX-2 (IC_50_ = 89.50 μg/mL), while showing higher selectivity for COX-2 [64].

The biological activity of compounds from the underground organs of *Eranthis* plants is actively being studied. Triterpene glycosides of the oleanane series found in *E. cilicica* tubers (compounds **34** and **35**, Table 1) have a cytotoxic effect on the HL-60 cell line, with average IC_50_ values of 10.5 µM [29]. The mechanism of the cytotoxicity may be related to their ability to induce apoptosis.

Extracts from representatives of *Eranthis* are expected to have antioxidant properties because they contain compounds with high antioxidant activity, e.g., flavonoids, phenolcarboxylic acids, and chromones. The antioxidant activity of chromones extracted from the tubers of *E. cilicica* has been evaluated against the superoxide anion radical in a chemiluminescent assay [21]. In that work, some chromone glycosides did not exert any obvious superoxide anion-scavenging ability, even at the sample’s concentration of 1000 μg/mL. Derivatives of 2-hydroxymethylchromone showed moderate activity (IC_50_ values were in the range of 179–274 μg/mL, respectively), which may be attributed to the presence of a 2-hydroxymethyl group. Epigallocatechin gallate, serving as a positive control, had an IC_50_ of 3.0 μg/mL [21].

The chromones found in *Eranthis* plants are of great interest to the pharmaceutical industry because they have high pharmacological activity but are not widespread in the plant kingdom. Some time ago, a study was conducted on the biosynthesis of individual chromones, for example, of khellol isolated from *E. hyemalis* [65]. The chemical synthesis of therapeutically active chromones from the plants of this genus is possible as well. For example, eranthin (compound **2**, Table 1) has already been synthesized artificially [66]. Investigation into the biosynthesis and subsequent chemical synthesis of such substances offers good prospects for obtaining new pharmacologically active compounds based on substances isolated from *Eranthis*.

Some papers on the lectin of *E. hyemalis* (EHL) [34,35,61] have shown that further research in this field is promising, as is the use of EHL for plant protection in studies on insecticidal, fungicidal, and bactericidal properties. EHL has an antiviral effect against alfalfa mosaic virus as well as a larvicidal effect against southern corn rootworm [35]. The research has been continued by a group led by McConnell [67], who have found that EHL impedes the development and reproduction of the nematode *Caenorhabditis elegans*. By conjugating EHL with gold nanoparticles, other researchers have obtained a drug called AuNPs@EHL, which is capable of inhibiting the reproduction of *C. elegans* [68].

*Eranthis* and some species of *Helleborus*—*H. niger* L., and *H. orientalis* Lam. (Ranunculaceae)—are often called poisonous plants because they contain cardiac glycosides [69,70]. Nonetheless, cardiac glycosides have not yet been found in *Eranthis* plants (see Section 2); these compounds are not detectable even in cardioactive fractions from *E. hyemalis* tubers [23,27,28]. It is likely that some other substances are simultaneously cardioactive and toxic in *Eranthis* plants. This phenomenon may be explained, for example, by the levels of chromones. Pharmacological trials of eranthin-β-D-gentiobioside (compound **5**, Table 1) have revealed a negative inotropic effect on an isolated guinea pig papillary muscle [23].

**Table 2:** Bioactive effects of *Eranthis* species and compounds isolated from them: preclinical studies (in vitro).

Species (Compounds)	Model/Method	Extracts	Dose or Result	References
Anti-inflammatory activity				
*E. hyemalis*	Inhibitory activity toward cyclooxygenase 1 (COX-1) and toward cyclooxygenase 2 (COX-2)	Ethanolic extract from roots	IC_50_ = 49.40 µg/mL IC_50_ = 89.50 µg/mL	[64]
Cytotoxic effect				
*E. cilicica* (3β-[(O-β-D-glucopyranosyl-(1→4)-O-[α-L-rhamnopyranosyl-(1→2)]-α-L-arabinopyranosyl) oxy]-23-hydroxyolean-12-en-28-oic acid; 3β-[(O-β-D-galactopyranosyl-(1→3)-O-α-L-rhamnopyranosyl-(1→2)-O-[β-D-glucopyranosyl-(1→4)]-α-L-arabinopyranosyl) oxy]-23-hydroxyolean-12-en-28-oic acid)	HL-60 cell line	Methanol extract of tubers	IC_50_ = 10.5 µM	[29]
Antioxidant activities				
*E. cilicica* (derivatives of 2-hydroxymethylchromone)	Superoxide anion radical in chemiluminescent assay	Methanol extract of tubers	IC_50_ values were in the range of 179–274 μg/mL	[21]
Antiviral activity				
*E. hyemalis* (lectin EHL)	Alfalfa mosaic virus	–	In the presence of EHL, alfalfa mosaic virus yielded 80–100% fewer lesions compared with alfalfa mosaic virus by itself	[35]
Larvicidal activity				
*E. hyemalis* (EHL)	Southern corn rootworm	–	Native EHL caused >90% mortality when fed to southern corn rootworm compared to control larvae	[35]
Anthelmintic activity				
*E. hyemalis* (EHL)	*C. elegans*	–	Impaired development and reproduction of *C. elegans*	[67,68]

Despite the limited geographic range of *Eranthis* plants and the small amount of plant raw material in existing populations, it is possible to search for active ingredients, develop methods for the biological and chemical synthesis of the isolated substances, and to create a finished therapeutic substance based on them.

## 4. Prospects for the Propagation of *Eranthis* Plants by Biotechnological Methods

*Eranthis* plants are geophytes, bloom in early spring, and—in addition to the abovementioned pharmacological potential—have high decorative value. Some representatives of this genus reproduce well vegetatively. For example, the *E. hyemalis* growing in Europe exhibits a high rate of tuber propagation and is cultivated widely [71]. This species is capable of self-renewal outside its range. Mayorov and Vinogradova [72] classify *E. hyemalis* as a potentially invasive species in the city of Moscow. Nevertheless, most representatives of *Eranthis* are endemics and have a limited geographic range. The cultivation of these plants in open ground is problematic. For instance, *E. sibirica* is considered unsuitable for introduction into Siberia [73,74]. The seed reproduction of *Eranthis* representatives is limited by the morphophysiological dormancy of seeds owing to underdeveloped embryos [75,76,77].

### 4.1. Introducing E. stellata into In Vitro Cultures

It is now possible to obtain the desired standardized, clean, uninfected plant material propagated in vitro. A protocol has been developed for introducing *E. stellata* into in vitro culture; this is an endemic species with limited occurrence in the Far East of Russia and in North Korea and Northeast China [78]. It has been revealed that peduncles with underdeveloped buds are the most efficient as the primary explants of *E. stellata*. It is reported that during introduction into in vitro cultivation, such explants should be cultivated at a low temperature (17 °C) in the dark to overcome phenolic oxidation. The half-strength Murashige–Skoog nutrient medium containing 5 μM 6-benzylaminopurine is reported to be optimal for this species’ propagation [78].

### 4.2. Implementation of E. tanhoensis Callus Cultures

Callus cultures can be employed for the sustainable large-scale production of secondary metabolites in the pharmaceutical, cosmetics, and food industries. On the basis of the callus cultures of medicinal plants, it is possible to produce phytochemicals for treating cancer and cardiovascular, neurodegenerative, and infectious diseases [79,80]. Conditions have been found for setting up and carrying out *E. tanhoensis* callus cultures [81]. For this purpose, those authors used etiolated tuber seedlings collected in the Mamai River Valley in Irkutsk Oblast (Siberia, Russia). Explants were cultured in test tubes containing the Murashige–Skoog agar medium supplemented with growth regulators: 2,4-dichlorophenoxyacetic acid (2 mg/L) and 6-benzylaminopurine (0.5 mg/L). Before transfer into the test tubes, sterile explants were immersed for 5 min in a liquid culture medium supplemented with 0.1 g/L ascorbic acid and 0.5 g/L polyvinylpyrrolidone. Two weeks after the planting, a thickening of the explants was observed, followed by callus formation. The resultant callus had a bright yellow color and dense consistency [81].

Further refinement of the protocol for the cultivation of *Eranthis* species in vitro and for the implementation of callus cultures of these plants will help to obtain large quantities of this material for preserving rare species and at the same time for using them in more in-depth phytochemical and pharmacological research.

## 5. Materials and Methods

The scientific literature was searched in various databases, including PubMed, Scopus, Google, Google Scholar, e-Library, and Web of Science, by means of the keyword “*Eranthis*”. All articles published in English from 1961 to August 2023 inclusively were found, as were articles in Russian, Chinese, Polish, Bulgarian, or German with an English abstract. Papers that have not addressed the phytochemistry, biological activity, and/or ethnopharmacology of *Eranthis* species were excluded. The chemical structures of the phytocomponents were found in the PubChem database, and the ChemDraw 19.0 software was used to draw selected structures.

## 6. Conclusions

The present review outlines the results of studies on the chemical composition and biological effects of species of the genus *Eranthis*. Our analysis of the literature data showed that there is information about the chemical profile and biological activity for only seven out of >14 *Eranthis* species of the world’s flora. In the extracts of the analyzed plants, more than 125 compounds have been identified: flavonoids, chromones, coumarins, phenolcarboxylic acids, and other classes of substances. For some stand-alone compounds, various biological activities have been demonstrated experimentally. Although *Eranthis* species have rarely been a subject of phytochemical and pharmacological investigation, the above information about their chemical composition and biological activity implies the fairly high potential usefulness of the medicinal properties of these species and justifies the interest in these plants among chemists studying new natural compounds. Furthermore, the analysis of the chemical composition of *Eranthis* species serves as a striking example of the fruitfulness of comprehensive research; this analysis helps to solve—inter alia—the taxonomic problems facing systematic botanists during studies on Ranunculaceae in general and *Eranthis* in particular.

## Figures and Tables

**Table 1:** Chemical constituents of the genus *Eranthis* (all classes of metabolites identified to date: vertical subdivisions in the table).

ID No.	Compound	Source *Eranthis* Species	Reference
Chromones			
1	8,11-dihydro-5-hydroxy-2,9-dihydroxymethyl-4H-pyrano [2,3-g][1] benzoxepin-4-one	*E. cilicica* (T *)	[21]
2	Eranthin (5-hydroxy-9-hydroxymethyl-2-methyl-8,11-dihydro-4H-pyrano[2,3-g][1]benzoxepin-4-one)	*E. hyemalis* (R)	[22]
3	Eranthin-β-D-glucoside (9-{[(β-D-glucopyranosvl)oxy]methyl}-8,11-dihydro-5-hydroxy-2-methyl-4H-pyrano[2,3-g][1]benzoxepin-4-one)	*E. hyemalis* (R, T)	[22,23]
4	Eranthin 9-β-D-glucopyranosyl-(1→6)-β-D-glucopyranoside	*E. cilicica* (T) *E. hyemalis* (T)	[21,23]
5	Eranthin β-D-gentiobioside (9-{[(β-D-gentiobiosyl)oxy]methyl}-8,11-dihydro-5-hydroxy-2-methyl-4H-pyrano[2,3-g][1]benzoxepin-4-one)	*E. hyemalis* (T)	[23]
6	2-C-Hydroxyeranthin β-D-glucopyranoside (9-{[(β-D-glucopyranosyl)oxy]methyl}-8,11-dihydro-5-hydroxy-2-(hydroxymethyl-4H-pyrano[2,3-g][1]benzoxepin-4-one)	*E. hyemalis* (T)	[23]
7	9-[(O-β-D-glucopyranosyl-(1→6)-β-D-glucopyranosyl)oxy]methyl-8,11-dihydro-5,9-dihydroxy-2-methyl-4H-pyrano[2,3-g][1]benzoxepin-4-one	*E. cilicica* (T)	[21]
8	8,11-dihydro-5,9-dihydroxy-9-hydroxymethyl-2-methyl-4H-pyrano[2,3-g][1]benzoxepin-4-one	*E. cilicica* (T)	[21]
9	5,7-dihydroxy-8-[(2E)-4-hydroxy-3-methylbut-2-enyl]-2-methyl-4H-1-benzopyran-4-one	*E. cilicica* (T)	[21]
10	5,7-dihydroxy-2-hydroxymethyl-8-[(2E)-4-hydroxy-3-methylbut-2-enyl]-4H-1-benzopyran-4-one	*E. cilicica* (T)	[21]
11	7-[(β-D-glucopyranosyl)oxy]-5-hydroxy-8-[(2E)-4-hydroxy-3-methylbut-2-enyl]-2-methyl-4H-1-benzopyran-4-one	*E. cilicica* (T)	[21]
12	7-[(β-D-glucopyranosyl)oxy]-5-hydroxy-2-hydroxymethyl-8-[(2E)-4-hydroxy-3-methylbut-2-enyl]-4H-1-benzopyran-4-one	*E. cilicica* (T)	[21]
13	7,8-Secoeranthin β-D-glucoside (8-{(2E)-4-[(β-D-glucopyranosyl)oxy]-3-methylbut-2-enyl}-5,7-dihydroxy-2-methyl-4H-1-benzopyran-4-one)	*E. hyemalis* (T)	[23]
14	2-C-Hydroxy-7,8-secoeranthin β-D-glucoside (8-{(2E)-4-[(β-D-glucopyranosyl)oxy]-3-methylbut-2-enyl}-5,7-dihydroxy-2-(hydroxymethyl)-4H-1-benzopyran-4-one)	*E. hyemalis* (T)	[23]
Furochromones			
15	Visnagin (4-methoxy-7-methyl-5H-furo[3,2-g]chromen-5-one)	*E. hyemalis* *E. longistipitata* (L)	[24,25]
16	Khellin (4,9-dimethoxy-7-methyl-5H-furo[3,2-g]chromen-5-one)	*E. hyemalis* *E. longistipitata* (L)	[24,25]
17	Khellol (7-(hydroxymethyl)-4-methoxyfuro[3,2-g]chromen-5-one)	*E. pinnatifida* (L, St)	[26]
18	Khellol glucoside (khellinin; 7-hydroxymethyl-4-methoxy-5H-furo [3,2-g]][1]benzopyran-5-one glucoside)	*E. hyemalis* (L, F)	[27]
19	Norkhellol (4-hydroxy-7-(hydroxymethyl)-5H-furo[3,2-g][1]benzopyran-5-one)	*E. pinnatifida* (L, St)	[26]
20	Norammiol (4-hydroxy-7(hydroxymethyl)-9-methoxy-5H-furo[3,2-g][1]-benzopyran-5-one)	*E. pinnatifida* (L, St)	[26]
21	7-[(O-β-D-glucopyranosyl-(1→6)-β-D-glucopyranosyl)oxy]methyl-4-hydroxy-5H-furo[3,2-g][1]benzopyran-5-one	*E. cilicica* (T)	[21]
22	Methoxsalen (9-methoxyfuro[3,2-g]chromen-7-one)	*E. longistipitata* (L)	[25]
23	Cimifugin (2S)-7-(hydroxymethyl)-2-(2-hydroxypropan-2-yl)-4-methoxy-2,3-dihydrofuro[3,2g]chromen-5-one)	*E. pinnatifida* (L, St) *E. cilicica* (T) *E. longistipitata* (L)	[21,25,26]
24	Norcimifugin (2S)-4-hydroxy-7-(hydroxymethyl)-2-(2-hydroxypropan-2-yl)-2,3-dihydrofuro[3,2-g]-chromen-5-one)	*E. pinnatifida* (L, St)	[26]
25	Cimifugin β-D-glucopyranoside (7-{[(β-D-glucopyranosy1)oxy]methyl}-2,3-dihydro-2-(l-hydroxy-1-methylethyl)-4-methoxy-5H-furo[3,2-g][1]benzopyran-5-one)	*E. hyemalis* (T)	[23]
26	5-O-Methylvisammioside (4-O-β-D-glucosyl-5-O-methylvisamminol)	*E. longistipitata* (L)	[25]
27	Visamminol-3′-O-glucoside (4-hydroxy-2-(2-hydroxypropan-2- yl)-methyl-2,3-dihydrofuro[3,2-g] chromen-5-one)	*E. longistipitata* (L)	[25]
Triterpene saponins			
28	Eranthisaponin A	*E. cilicica* (T)	[28]
	(3β-[(O-β-D-allopyranosyl-(1→3)-O-α-L-rhamnopyranosyl-(1→2)-O-[β-D-glucopyranosyl-(1→4)]-α-L-arabinopyranosyl)oxy]-23-hydroxyolean-12-en-28-oic acid 28-O-α-L-rhamnopyranosyl-(1→4)-O-β-D-glucopyranosyl- (1→6)-β-D-glucopyranoside)		
29	Eranthisaponin B (3β-[(O-β-D-glucopyranosyl-(1→4)-O-[α-L-rhamnopyranosyl-(1→2)]-α-L-arabinopyranosyl) oxy]-23-hydroxyolean-12-en-28-oic acid 28-O-α-L-rhamnopyranosyl-(1→4)-O-β-D-glucopyranosyl-(1→6)-O-β-D-glucopyranosyl-(1→4)-O-α-L-rhamnopyranosyl-(1→4)-O-β-D-glucopyranosyl- (1→6)-β-D-glucopyranoside)	*E. cilicica* (T)	[28]
30	3β-[(O-β-D-glucopyranosyl-(1→4)-O-[α-L-rhamnopyranosyl-(1→2)]-α-L-arabinopyranosyl) oxy]-23-hydroxyolean-12-en-28-oic acid 28-O-α-L-rhamnopyranosyl-(1→4)-O-β-D-glucopyranosyl-(1→6)-β-D-glucopyranoside	*E. cilicica* (T)	[28]
31	23-Hydroxy-3β-[(O-α-L-rhamnopyranosyl-(1→2)-α-L-arabinopyranosyl)oxy]olean-12-en-28-oic acid 28-O-α-L-rhamnopyranosyl-(1→4)-O-β-D-glucopyranosyl-(1→6)-β-D-glucopyranoside	*E. cilicica* (T)	[28]
32	3β-[(O-β-D-glucopyranosyl-(1→4)-α-L-arabinopyranosyl)oxy]-23-hydroxyolean-12-en-28-oic acid 28-O-α-L-rhamnopyranosyl-(1→4)-O-β-D-glucopyranosyl-(1→6)-β-D-glucopyranoside	*E. cilicica* (T)	[28]
33	3β-[(O-β-D-glucopyranosyl-(1→2)-O-[β-D-glucopyranosyl-(1→4)]-α-L-arabinopyranosyl)oxy]-23-hydroxyolean-12-en-28-oic acid 28-O-α-L-rhamnopyranosyl-(1→4)-O-β-D-glucopyranosyl-(1→6)-β-D-glucopyranoside	*E. cilicica* (T)	[28]
34	3β-[(O-β-D-glucopyranosyl-(1→4)-O-[α-L-rhamnopyranosyl-(1→2)]-α-L-arabinopyranosyl) oxy]-23-hydroxyolean-12-en-28-oic acid	*E. cilicica* (T)	[29]
35	3β-[(O-β-D-galactopyranosyl-(1→3)-O-α-L-rhamnopyranosyl-(1→2)-O-[β-D-glucopyranosyl-(1→4)]-α-L-arabinopyranosyl) oxy]-23-hydroxyolean-12-en-28-oic acid	*E. cilicica* (T)	[29]
36	(23R,24R,25R)-16β,23:23,26:24,25-triepoxy-28-hydroxy-9,19-cycloartan-3β-yl β-D-glucopyranoside	*E. cilicica* (T)	[29]
37	(23R,24R,25R)-16β,23:23,26:24,25-triepoxy-9,19-cycloartane-3β,28-diol	*E. cilicica* (T)	[29]
38	(23R,24R,25R)-16β,23:23,26:24,25-triepoxy-28-hydroxy-9,19-cylcoartan-3β-yl O-β-D-glucopyranosyl-(1→4)-β-D-glucopyranoside	*E. cilicica* (T)	[29]
39	(23R,24R,25R)-16β,23:23,26:24,25-triepoxy-28-hydroxy-9,19-cycloartan-3β-yl O-β-D-glucopyranosyl-(1→3)-β-D-glucopyranoside	*E. cilicica* (T)	[29]
40	(23R,24R,25R)-16β,23:23,26:24,25-triepoxy-28-hydroxy-9,19-cycloartan-3β-yl O-β-D-glucopyranosyl-(1→6)-β-D-glucopyranoside	*E. cilicica* (T)	[29]
41	(23R,24R,25R)-16β,23:23,26:24,25-triepoxy-28-hydroxy-9,19-cylcoartan-3β-yl O-β-D-glucopyranosyl-(1→4)-O-[β-D-glucopyranosyl (1→6)]-β-D-glucopyranoside	*E. cilicica* (T)	[29]
42	(23R,24R,25R)-16β,23:23,26:24,25-triepoxy-28-oxo-9,19-cycloartan-3β-yl O-β-D-glucopyranosyl-(1→4)-β-D-glucopyranoside	*E. cilicica* (T)	[29]
43	(23R,24R,25R)-16β,23:23,26:24,25-triepoxy-9,19-cycloartan-3β-yl O-β-D-glucopyranosyl-(1→6)-β-D-glucopyranoside	*E. cilicica* (T)	[29]
44	(23S,24R,25R)-16β,23:23,26:24,25-triepoxy-28-hydroxy-9,19-cycloartan-3β-yl O-β-D-glucopyranosyl-(1→4)-β-D-glucopyranoside	*E. cilicica* (T)	[29]
45	(23S,24R,25R)-16β,23:23,26:24,25-triepoxy-9,19-cycloartan-3β,28-diol	*E. cilicica* (T)	[29]
46	(23S,24R,25R)-16β,23:23,26:24,25-triepoxy-28-hydroxy-9,19-cylcoartan-3β-yl O-β-D-glucopyranosyl-(1→3)-β-D-glucopyranoside	*E. cilicica* (T)	[29]
47	(23S,24R,25R)-16β,23:23,26:24,25-triepoxy-28-hydroxy-9,19-cylcoartan-3β-yl O-β-D-glucopyranosyl-(1→6)-β-D-glucopyranoside	*E. cilicica* (T)	[29]
48	(23S,24R,25R)-16β,23:23,26:24,25-triepoxy-9,19-cycloartan-3β-yl O-β-D-glucopyranosyl-(1→6)-β-D-glucopyranoside	*E. cilicica* (T)	[29]
Alkaloids			
49	Corytuberine (2,10-dimethoxy-6α-aporphine-1,11-diol)	*E. hyemalis* (T; Ap)	[30]
Coumarins			
50	5,7-Dihydroxy-4-methylcoumarin	*E. longistipitata* (L)	[25]
51	Scoparone (6,7-dimethoxycoumarin)	*E. longistipitata* (L)	[25]
52	Fraxetin (7,8-dihydroxy-6-methoxycoumarin)	*E. longistipitata* (L)	[25]
53	Luvangetin (10-methoxy-2,2-dimethylpyrano[3,2-g]chromen-8-one)	*E. longistipitata* (L)	[25]
Flavonoids			
54	Quercetin	*E. longistipitata* (L) *E. stellata* (L) *E. tanhoensis* (L)	[25,31,32]
55	Isoquercitrin (quercetin-3-O-β-D-glucoside)	*E. longistipitata* (L)	[25,32]
56	Hyperoside (quercetin 3-O-β-D-galactoside)	*E. longistipitata* (L)	[25,32]
57	Reynoutrin (quercetin-3-O-β-D-xylopyranoside)	*E. longistipitata* (L)	[25,32]
58	Quercetin-6-O-β-D-xylopyranosyl-β-D- glucopyranoside	*E. longistipitata* (L)	[25,32]
59	Quercetin-3-sambubioside (quercetin-3-O-[β-D-xylosyl-(1→2)-β-D-glucoside])	*E. longistipitata* (L)	[25,32]
60	Peltatoside (quercetin-3-(6-O-α-L-arabinopyranosyl)-β-D- glucopyranoside))	*E. longistipitata* (L)	[25,32]
61	Rutin (quercetin 3-O-β-D-rutinoside)	*E. longistipitata* (L)	[25,32]
62	Kaempferol	*E. longistipitata* (L) *E. stellata* (L) *E. tanhoensis* (L)	[25,31,32]
63	Juglalin (kaempferol 3-O-α-L-arabinopyranoside)	*E. longistipitata* (L)	[25,32]
64	Trifolin (kaempferol-3-O-β-D-galactoside)	*E. longistipitata* (L)	[25,32]
65	Aromadendrin [(+)-dihydrokaempferol]	*E. longistipitata* (L)	[25,32]
66	Vitexin (apigenin 8-C-glucoside)	*E. sibirica* (L)	[31]
67	Orientin (luteolin-8-C-glucoside)	*E. sibirica* (L)	[31]
		*E. stellata* (L)	
68	Carlinoside (luteolin 6-C-β-D-glucopyranoside-8-C-α-L-	*E. longistipitata* (L)	[25,32]
	arabinopyranoside)		
69	Cianidanol [(+)-catechin]	*E. longistipitata* (L)	[25,32]
70	Auriculoside (7,3,5′-trihydroxy-4′-methoxyflavan-3′-glucoside)	*E. longistipitata* (L)	[25,32]
71	6-Methoxytaxifolin	*E. longistipitata* (L)	[25,32]
72	Aspalathin	*E. longistipitata* (L)	[25,32]
73	Phloridzin (phloretin-2′-O-β-glucoside)	*E. longistipitata* (L)	[25,32]
74	Phloretin (dihydroxy naringenin)	*E. longistipitata* (L)	[25,32]
Cinnamic acids			
75	Chlorogenic acid (3-O-caffeoylquinic acid)	*E. sibirica* (L) *E. stellata* (L) *E. tanhoensis* (L)	[31]
76	Caffeic acid (3,4-dihydroxycinnamic acid)	*E. sibirica* (L) *E. stellata* (L)	[31]
Phenolic acids			
77	Salicylic acid (3-tert-2-butyl-2-hydroxy-6-methylbenzoic acid)	*E. sibirica* (L) *E. tanhoensis* (L)	[31]
78	Gentisic acid (2,5-dihydroxybenzoic acid)	*E. stellata* (L)	[31]
Fatty acids and their derivatives			
79	Myristic acid (14:0)	*E. hyemalis* (S)	[33]
80	Pentadecylic acid (15:0)	*E. hyemalis* (S)	[33]
81	Palmitic acid (16:0)	*E. hyemalis* (S)	[33]
82	16-Hydroxyhexadecanoic acid	*E. longistipitata* (L)	[25]
83	cis-5-Hexadecenoic acid (16:1 Δ5cis)	*E. hyemalis* (S)	[33]
84	Palmitoleic acid (16:1 Δ9cis)	*E. longistipitata* (L)	[25]
85	cis-9-Octadecanoic acid (18:0 Δ9cis)	*E. hyemalis* (S)	[33]
86	cis-Vaccenic acid (18:1 Δ11cis)	*E. hyemalis* (S)	[33]
87	Linoleic acid (18:2 Δ9cis, 12cis)	*E. hyemalis* (S)	[33]
88	9-oxo-ODA (9-Oxo-trans-10, trans-12-octadecadienoic acid)	*E. longistipitata* (L)	[25]
89	(+/−)13-HODE	*E. longistipitata* (L)	[25]
	(13-hydroxyoctadecadienoic acid)		
90	Corchorifatty acid F (9,12,13-trihydroxy-10(E),15(Z)-octadecadienoic acid)	*E. longistipitata* (L)	[25]
91	α-Linolenic acid (18:3 Δ9cis, 12cis, 15cis)	*E. hyemalis* (S) *E. longistipitata* (L)	[25,33]
92	Linolenic acid ethyl ester	*E. longistipitata* (L)	[25]
93	α-Eleostearic acid (18:3 Δ9cis, 11trans, 13trans)	*E. longistipitata* (L)	[25]
94	Pinolenic acid (18:3 Δ5cis, 9cis, 12cis)	*E. longistipitata* (L)	[25]
95	13(S)-HOTrE (13-OH-cis-9, trans-11, cis-15-octadecatrienoic acid)	*E. longistipitata* (L)	[25]
96	(15Z)-9,12,13-Trihydroxy-15-octadecenoic acid	*E. longistipitata* (L)	[25]
97	12-Oxo-phytodienoic acid	*E. longistipitata* (L)	[25]
98	9S,13R-12-Oxo-phytodienoic acid	*E. longistipitata* (L)	[25]
99	Arachidic acid (20:0)	*E. hyemalis* (S)	[33]
100	cis-5-Eicosenoic acid (20:1 Δ5cis)	*E. hyemalis* (S)	[33]
101	Gondoic acid (20:1 Δ11cis)	*E. hyemalis* (S)	[33]
102	Keteleeronic acid (20:2 Δ5cis, 11cis)	*E. hyemalis* (S)	[33]
103	cis-11,14-Eicosadienoic acid (20:2 Δ11cis, 14cis)	*E. hyemalis* (S)	[33]
104	15-OxoEDE (15-Oxo-cis-11,trans-13-eicosadienoic acid)	*E. longistipitata* (L)	[25]
105	cis-5,11,14-Eicosatrienoic acid (20:3 Δ5cis, 11cis, 14cis)	*E. hyemalis* (S)	[33]
106	Behenic acid (22:0)	*E. hyemalis* (S)	[33]
107	Erucic acid (22:1 Δ13cis)	*E. hyemalis* (S)	[33]
108	cis-5,13-Docosadienoic acid (22:2 Δ5cis, 13cis)	*E. hyemalis* (S)	[33]
109	cis-13,16-Docosadienoic acid (22:2 Δ13cis, 16cis)	*E. hyemalis* (S)	[33]
110	cis-5,13,16-Docosatrienoic acid (22:3 Δ5cis, 13cis, 16cis)	*E. hyemalis* (S)	[33]
111	cis-10,13,16-Docosatrienoic acid (22:3 Δ10cis, 13cis, 16cis)	*E. hyemalis* (S)	[33]
Amino acids			
112	D-(+)-Pyroglutamic acid	*E. longistipitata* (L)	[25]
113	D-(+)-Tryptophan	*E. longistipitata* (L)	[25]
114	Isoleucine	*E. longistipitata* (L)	[25]
115	L-Phenylalanine	*E. longistipitata* (L)	[25]
116	L-Tyrosine	*E. longistipitata* (L)	[25]
117	D-(−)-Glutamine	*E. longistipitata* (L)	[25]
Organic acids			
118	Citric acid	*E. longistipitata* (L)	[25]
119	D-α-Hydroxyglutaric acid	*E. longistipitata* (L)	[25]
120	Gluconic acid	*E. longistipitata* (L)	[25]
Sugars			
121	α-Lactose	*E. longistipitata* (L)	[25]
122	D-(+)-Galactose	*E. longistipitata* (L)	[25]
123	α.α-Trehalose	*E. longistipitata* (L)	[25]
Alcohols			
124	D-(−)-Mannitol	*E. longistipitata* (L)	[25]
Phenylpropanoids			
125	6-Gingerol	*E. longistipitata* (L)	[25]
Lectins			
126	EHL	*E. hyemalis* (R)	[34,35]

* Ap, aerial part; F, flowers; L, leaves; R, rhizome; S, seeds; St, stems; T, tubers.

## Data Availability

Not applicable.
